# Does lymphadenectomy predict survival in early-stage epithelial ovarian cancer? An updated systematic review and meta-analysis

**DOI:** 10.12669/pjms.41.8.12365

**Published:** 2025-08

**Authors:** Yan Jin, Chaoyou Zhou

**Affiliations:** 1 Yan Jin Department of Gynaecology, Huzhou Maternity & Child Health Care Hospital, Huzhou City, Zhejiang Province 313000, P.R. China; 2 Chaoyou Zhou Department of Gynaecology, Huzhou Maternity & Child Health Care Hospital, Huzhou City, Zhejiang Province 313000, P.R. China

**Keywords:** Lymph node dissection, Ovarian carcinoma, Staging, Mortality, Relapse

## Abstract

**Background & Objective:**

The added value of lymphadenectomy is a widely debated topic, especially in cases of early-stage epithelial ovarian cancer (EOC). We present robust evidence through an updated systematic review and meta-analysis regarding the effects of lymphadenectomy on overall survival (OS) and progression-free survival (PFS) in early-stage EOC (eEOC).

**Methods:**

PubMed, PubMed CENTRAL, Embase, and Web of Science databases were searched from their inception until 10 March 2025 for all types of studies reporting adjusted outcomes of eEOC based on lymphadenectomy. Random-effect meta-analysis, subgroup analysis, and meta-regression were conducted.

**Results:**

Twenty-four studies were included. The number of studies was far more than previously published reviews (4-8 studies). Meta-analysis of 20 and 13 studies showed that patients undergoing lymphadenectomy had significantly better OS (HR: 0.808 95% CI: 0.692, 0.943 I^2^=34%) and PFS (HR: 0.743 95% CI: 0.583, 0.947 I²=44%) respectively. The estimates were stable on exclusion of majority studies on sensitivity analysis. Meta-regression showed that lymph node metastasis in the lymphadenectomy group and use of chemotherapy in the lymphadenectomy and control groups did not have a significant impact on the results. Subgroup analysis based on study type, location, histology, stage, protocols of study and control groups, and adjustment for chemotherapy and cancer stage in the multivariate analysis of the studies showed mixed results.

**Conclusions:**

Lymphadenectomy in eEOC may lead to better OS and PFS. The retrospective nature of the data and the heterogeneity among studies are significant limitations that warrant caution in interpretation of the results.

***Registration No.:*** PROSPERO (CRD420250656184).

## INTRODUCTION

Ovarian cancer is among the most deadly gynecological cancers globally. It ranks as the second most prevalent genital malignancy and is the leading cause of genital cancer-related mortality among females.^1^ Ovarian cancer affects not only survival rates but also significantly diminishes overall quality of life and sexual function in those impacted.^2^ Epithelial ovarian cancer (EOC) is the most common histological type, representing over 85% of all ovarian cancer cases.^3^ These tumors are further classified into serous, endometrioid, clear cell, and mucinous carcinoma, each exhibiting distinct differences in etiology, morphology, molecular biology, and prognosis.^4^

Ovarian cancer is typically diagnosed at an advanced stage; however, around 23% of patients are identified at an early stage, specifically categorized as FIGO (International Federation of Obstetrics and Gynecology) stages I and II. Their five years survival rate is significantly greater as compared to individuals with advanced disease.^5^ Comprehensive staging surgery, which encompasses systematic pelvic and para-aortic lymphadenectomy, is the standard procedure for early-stage EOC (eEOC).^6^,^7^ Lymphadenectomy primarily serves to accurately define the stage, which has significant clinical ramifications for the future. Patients are upstaged to IIIA if histologically confirmed positive nodes are found in patients earlier thought to have early-stage disease.^6^ Prior research indicates that the incidence of lymph node invasion in patients with clinically apparent early-stage ovarian cancer ranges from 5.1% to 20%, leading to high rates of overtreatment (in about 80% cases).^8^-^10^ Moreover, performing a systematic pelvic and para-aortic lymphadenectomy is difficult and may carry a significant risk of adverse outcomes during and after surgery.^11^

In cases of advanced ovarian cancer, the recently concluded LION study, which compared lymphadenectomy with no-lymphadenectomy, showed that lymphadenectomy had no appreciable positive impact on overall survival (OS) and progression-free survival (PFS). Conversely, the lymphadenectomy group exhibited a significant increase in serious complications, including early mortality and reintervention.^12^ The solitary randomized controlled trial (RCT)^13^ assessing the effects of lymphadenectomy for eEOC also found no significant difference in OS and PFS between the study groups, despite an overall effect size favoring the lymphadenectomy group (OS, HR: 0.85; PFS, HR: 0.72). Additionally, because of the small number of cases, the RCT lacked the statistical ability to identify a difference in survival. The inconsistency between the results of the LION study^12^ (no survival benefit of lymph node dissection in advanced ovarian cancer) and the results of the eEOC study^13^ show that the role of lymphadenectomy in ovarian cancer remains highly controversial.

Several retrospective studies^10^,^14^-^16^ have also examined the relationship between lymphadenectomy and survival in eEOC patients; however, a consensus has not been achieved due to the varying results across different studies. Two previous meta-analyses^17^,^18^ aimed at generating high-quality evidence on the subject included only four to eight studies, thereby constraining the statistical power of the findings for definitive conclusions. There is currently no authoritative conclusion based on large-scale retrospective data and subgroup analysis, and updated evidence is urgently needed to guide clinical decision-making. Given these limitations, this review was undertaken to present the most current and thorough evidence regarding the impact of lymphadenectomy on OS and PFS in eEOC.

## METHODS

This systematic review was prospectively registered in PROSPERO (CRD420250656184) and reported in accordance with the 2020 Preferred Reporting Items for Systematic Reviews and Meta-Analyses statement.^19^ No patient contact or intervention was required for the study, so approval from our institutional review board was not necessary. Two authors conducted independent searches of PubMed, CENTRAL, Embase, and Web of Science from their inception until 10 March 2025. The search methodology was constructed with a mixture of free-text and MeSH keywords. The following search terms were used: “ovarian neoplasm,” “ovarian cancer,” “ovarian carcinoma,” “lymphadenectomy,” “lymph node dissection,” “lymph node excision,” “survival,” “mortality,” “death,” “relapse,” “recurrence,” “PFS,” “OS,” “RFS,” and “DFS” in conjunction with the Boolean operators AND/OR in order to find any potential studies that evaluated the results of EOC based on lymphadenectomy. Details of the search strategy pertaining to each database are shown in Supplementary Table-I. To enhance the sensitivity of the search procedure, we manually examined the reference lists of the first obtained papers to identify additional eligible publications.

**Supplementary Table-I:** Search strategy of databases. PubMed

Search	Query
#1	("lymph node dissection"[All Fields] OR "lymph node excision"[MeSH Terms] OR "lymph node excision"[All Fields] OR "lymphadenectomies"[All Fields] OR "lymphadenectomy"[All Fields])
#2	("ovarian neoplasms"[MeSH Terms] OR "ovarian neoplasms"[All Fields] OR "ovarian cancer"[All Fields] OR "ovarian carcinoma"[All Fields] OR "ovary carcinoma"[All Fields] OR "ovary cancer"[All Fields] OR "ovary neoplasm"[All Fields])
#3	"mortality"[MeSH Terms] OR "mortality"[All Fields] OR "death"[MeSH Terms] OR "death"[All Fields] OR OR "survival"[MeSH Terms] OR "survival"[All Fields] "OS"[All Fields] OR "PFS"[All Fields] OR "RFS"[All Fields] OR "recurrence"[All Fields] "replase"[All Fields]
#4	#1 AND #2 AND #3

**Table:** Embase

Search	Query
#1	(’lymph node dissection’/exp OR ’lymph node dissection’ OR ’lymphadenectomy’/exp OR lymphadenectomy OR ’lymph node excision’/exp OR ’lymph node excision’)
#2	(’ovarian neoplasms’/exp OR ’ovarian neoplasms’ OR ’ovarian cancer’/exp OR ’ovarian cancer’ OR ’ovarian carcinoma’/exp OR ’ovarian carcinoma’ OR ’ovary carcinoma’/exp OR ’ovary carcinoma’ OR ’ovary cancer’/exp OR ’ovary cancer’ OR ’ovary neoplasm’/exp OR ’ovary neoplasm’)
#3	(’mortality’/exp OR mortality:ab,ti OR ’death’/exp OR death:ab,ti OR ’survival’/exp OR survival:ab,ti OR os:ab,ti OR pfs:ab,ti OR rfs:ab,ti OR ’recurrence’/exp OR recurrence:ab,ti OR ’relapse’/exp OR relapse:ab,ti)
#4	#1 AND #2 AND #3

**Table:** Web of Science

Search	Query
#1	ALL FIELDS: (("lymph node dissection") OR (Lymphadenectomy) OR (Lymph Node Excision))
#2	ALL FIELDS: ("ovarian neoplasms" OR "ovarian cancer" OR "ovarian carcinoma" OR "ovary carcinoma" OR "ovary cancer" OR "ovary neoplasm")
#3	ALL FIELDS: (mortality OR death OR survival OR OS OR PFS OR RFS OR recurrence OR relapse)
#4	#1 AND #2 AND #3

**Table:** CENTRAL

Search	Query
#1	MeSH descriptor: [Lymph Node Excision] explode all trees
#2	("lymph node dissection" OR "lymph node excision" OR "lymphadenectomies" OR "lymphadenectomy"):ti,ab,kw (Word variations have been searched)
#3	#1 OR #2
#4	MeSH descriptor: [Ovarian Neoplasms] explode all trees
#5	("ovarian neoplasms" OR "ovarian cancer" OR "ovarian carcinoma" OR "ovary carcinoma" OR "ovary cancer" OR "ovary neoplasm"):ti,ab,kw (Word variations have been searched)
#6	#4 OR #5
#7	MeSH descriptor: [Mortality] explode all trees
#8	MeSH descriptor: [Death] explode all trees
#9	MeSH descriptor: [Survival] explode all trees
#10	(mortality OR death OR surviv* OR OS OR PFS OR RFS OR recurrence* OR relapse):ti,ab,kw (Word variations have been searched)
#11	#7 OR #8 OR #9 OR #10
#12	#3 AND #6 AND #11
#13	#12 in Trials

Following the elimination of duplicate studies, the abstracts were independently evaluated by two writers for potentially relevant research. Any subsequent disputes were adjudicated by consensus. Following the exclusion of nonrelevant studies through abstract screening, a comprehensive full-text examination of the remaining abstracts was performed to ensure completeness and eligibility of the presented data, in accordance with the predefined selection criteria.

### Pre-defined selection criteria:

Predefined search criteria were created according to PICOS. The specifics of each part were as follows:

*Population:* individuals diagnosed with early-stage epithelial ovarian carcinoma (EOC).

*Intervention:* Lymphadenectomy or comprehensive staging.

*Comparison:* No lymphadenectomy, lymph node sampling only, or clinical lymph node assessment.

*Outcomes:* Studies were required to report OS or PFS.

*Study classification:* All categories, encompassing observational studies and RCT.

We excluded studies not in English or Chinese language, studies on combined early and advanced EOC, studies not reporting adjusted summary estimates of OS or PFS, studies without a control group, studies with <20 patients, and studies only available as abstracts. Studies from the same database were accepted for the review provided they reported different outcomes or different histological subtypes of EOC, otherwise they were excluded.

### Quality assessment:

Two researchers evaluated the quality of observational studies with the Newcastle Ottawa Scale (NOS).^20^ The NOS assesses research for cohort representativeness, comparability, and outcome measurement. Each of these is assigned scores of 0-4, 0-2, and 0-3, respectively. The quality of the included RCTs was assessed using the risk of bias-2 tool of the Cochrane collaboration.^21^ Discrepancies among reviewers were resolved through dialogue.

### Data extraction:

Details of first author, year, study design, type of EOC, FIGO stage, protocol of lymphadenectomy and control groups, sample size, age, use of adjuvant chemotherapy (AC), lymph node metastasis in lymphadenectomy group, factors adjusted for reporting outcomes, hazard ratio (HR) and 95% confidence intervals (CI) of OS and PFS, and follow-up were obtained from every study by two reviewers. Pre-defined outcomes were OS and PFS. Articles with missing data were not included and no data assumptions were made.

### Data analysis:

We utilized “Comprehensive Meta-Analysis” (version-3) for the meta-analyses. An aggregated estimate of OS and PFS was produced by pooling HR of individual studies. The most extensively adjusted summary estimate was utilized if multiple estimates were reported by the studies. A random-effects meta-analysis was conducted, and statistical heterogeneity was evaluated using the I² statistic, which signifies low heterogeneity for I² < 25%, moderate heterogeneity for I² between 25% and 75%, and high heterogeneity for I² > 75%. Egger’s test and funnel plots were employed to assess publication bias. Sensitivity analysis to check for the impact of each study on the pooled summary estimate was conducted. Subgroup analysis was performed based on study type, location, histology, stage, protocols of study and control groups, and adjustment for AC and stage in the multivariate analysis of the studies. Random-effects meta-regression analysis was performed to examine the effect of the following continuous variables on OS and PFS: lymph node metastasis in the lymphadenectomy group and use of AC in the lymphadenectomy and control groups.

## RESULTS

### Database search outcomes:

**Fig.1:**
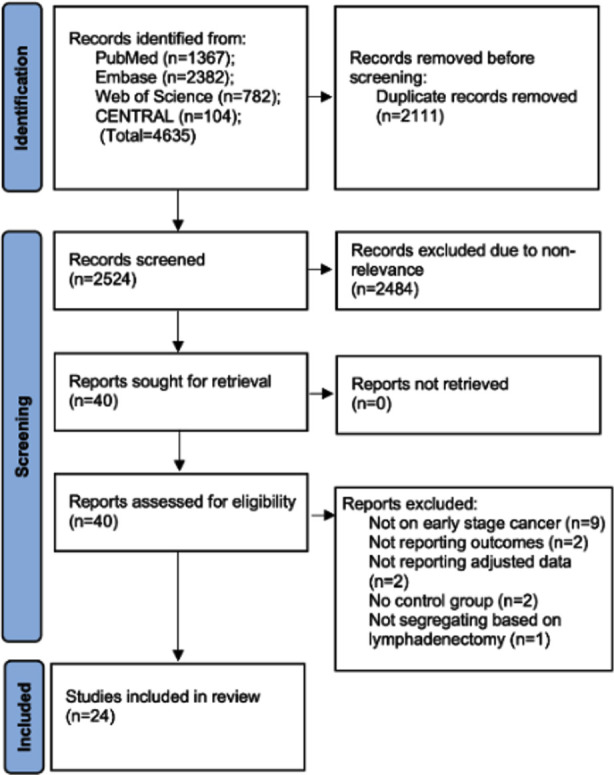
PRISMA flowchart.

Fig.1 illustrates the search hits documented by the reviewers at each stage of the research selection process. A total of 4635 studies were initially found, of which 2524 were unique. Forty studies were identified as eligible for thorough examination after the initial screening. Twenty-four studies^10^,^13^-^16^,^22-40^ were included in the review. We observed a strong concordance among the reviewers regarding research inclusion (kappa=0.89).

### Study information:

Table-I and Supplementary Table-II displays the data that the authors were able to gather. One study was an RCT,^13^ one was a prospective cohort study^34^ and the remaining studies were retrospective. Data was from Asia, Europe, or North America. The bulk of studies provided data on mixed EOC, whereas a subset focused primarily on clear cell, serous, mucinous, and endometrioid subtypes. The majority of studies included stage I or stage I-II disease. Three studies further included stage III EOC, but the overall percentage of stage III cases was <10% of the sample. Two articles by Bizzari et al^14^,^37^ were included, featuring overlapping data.

**Table-I:** Details of included studies.

Study Study design Country	Ovarian cancer histology	FIGO stage	Nodes removed in lymphadenectomy group	Median no of nodes removed in lymphadenectomy group	Groups	Sample size	Age	Adjuvant chemotherapy (%)	Lymph node metastasis	Median follow-up
Abe 2010[36] R Japan	Mixed	I-III (stage III: 6.5%)	Pelvic and /or para-aortic lymph nodes	9-80	L NL	40 22	55.5 (43,72) 45.1 (20,63)	97.3 82.6	10	Median 31 months
Bao 2024[16] R China	Mixed	IA-IIA	Pelvic and /or para-aortic lymph nodes	NR	L NL	242 42	55± 12.3 52.26± 13.9	NR	3.3	Median 63 months
Bizzari 2021[37] R Italy and Germany	Mixed	IA-IIIA1 (stage III: 9.4%)	Pelvic and para-aortic lymph nodes	32 (1-149)	L NL	360 129	54±10.6 60±12.1	100 100	12.8	Median 63 (5-342) months
Bizzarri 2023[14] R Germany and Italy	Endometrioid	IA-IIIA1 (stage III: 3.7%)	Pelvic and /or para-aortic lymph nodes	NR	L NL	199 99	49 (43-58) 56 (47.5-62)	47.7 44.4	5.5	Median 45 months
Chen 2021*[38] R United States	Mixed	I	Pelvic and /or para-aortic lymph nodes	NR	L NL	581 581	NR	35.3 35.6	3.9	NR
Chen 2022[39] R China	Serous	I-II	Pelvic and /or para-aortic lymph nodes	NR	L NL	67^	NR	NR	NR	NR
Deng 2021[10] R China	Mixed	I-II	Pelvic and para-aortic lymph nodes	25(NR)	L NL	319 81	47 47	90.6 86.4	3.1	Median 69 (4-195) months
Ignatov 2022[40] R Germany	NR	T1a-2a	Pelvic and para-aortic lymph nodes	33 (12-96)	L NL	168 131	59 (21-82) 64 (25-92)	83.3 78.6	10.2	Median 51 (1-209) months
Kim 2023[22] R Canada	Mucinous	I	Pelvic and /or para-aortic lymph nodes	NR	L NL	48 101	49 (33-58)^	10^	2	Median 3.9 (2.1- 5.6) years
Liu 2024*[23] R China	Clear cell	I-II	Pelvic and /or para-aortic lymph nodes	NR	L NL	121 22	NR	58.7 50	NR	Median 38 (24-61) months
Maggioni 2006[13] RCT Italy	Mixed	I-II	Pelvic and para-aortic lymph nodes. Random sampling in NL group	47 (33-63)	L NL	138 130	51 (43-60) 52 (44-59)	56 66	22	Median 87.8 (62.7- 120.6) months
Matsuo 2018[15] R USA	Mixed	I-II	> 12 pelvic lymph nodes removed. Inadequate sampling in NL group	11 (NR)	L NL	8489 4628	56^	NR	NR	Median 7.1 years
Michel 2023[24] R France	Mixed	I-II	Pelvic and/or para-aortic lymph nodes	11(1-69)	L NL	98 64	56.3±13.5 67.7±14.4	69.4 43.8	5.1	Median 8.4 years
Miyamoto 2023*[25] R Japan	Mixed	IA	Pelvic and para-aortic lymph nodes	NR	L NL	117 176	54.32± 10.2 52.41± 15.9	47.9 44.9	NR	Median 5.9 years
Nasioudis 2019[26] R USA	Mucinous	I	Pelvic and /or para-aortic lymph nodes	NR	L NL	3367 1444	51 (NR)^	45.3	NR	Median 57.8- 63.11
Oshita 2013[27] R Japan	Mixed	I-II	Pelvic and para-aortic lymph nodes	34 (20-52)	L NL	284 138	53.5 (17-80) 52 (16-91)	87.3 68.1	8.1	Median 64.9 (42.3, 90.8) months
Sun 2025[28] R China	Endometrioid	I	NR	NR	L NL	88 28	43.4± 10 45.9± 10.2	83 71.4	NR	NR
Svolgaard 2014[29] P Denmark	Mixed	I	Pelvic and /or para-aortic lymph nodes	NR	L NL	216 411	59^	NR	6	Median 38 (1-76) months
Wang 2024[30] R China	Mixed	I-II	NR	NR	L NL	217 98	50.67±9.2 50.96±12.3	19.4 19.4	NR	Median 88 (60-156) months
Yamazaki 2018[31] R Japan	Clear cell	I-II	Pelvic and para-aortic lymph nodes. Clinical evaluation and/or pelvic lymphadenectomy in NL group	59	L NL	79 48	53.9± 10.1 53.8± 9.3	NR	12	NR
Yang 2023[32] R South Korea	Mixed	I-II	Pelvic and/or para-aortic lymph nodes	21	L NL	453 133	52 [46-58] 52[41-62]	84.1 66.1	3.1	Median 44 (3-143) months
Yoshihara 2020*[33] R Japan	Mucinous	I	Pelvic and para-aortic lymph nodes; Clinical evaluation and/or pelvic lymphadenectomy in NL group	NR	L NL	55 131	50.7± 11.4 46.4± 17.6	59.6 57.7	1.8	NR
Yoshihara 2021*[34] R Japan	Endometrioid	I	Pelvic and para-aortic lymph nodes; Clinical evaluation and/or pelvic lymphadenectomy in NL group	NR	L NL	145 114	53± 9.5 53.4±14.6	80.6 69.7	2.8	NR
Zhao 2017[35] R China	Endometrioid	I	Pelvic and /or para-aortic lymph nodes	18 (2-48)	L NL	72 6	48.37± 13.3^	89.7^	NR	Median 74.5 (56-117) months

NR, not reported; R, retrospective; P, prospective; RCT, randomized controlled trial; L, lymphadenectomy; NL, non-lymphadenectomy group; OS, overall survival; PFS, progression-free survival; FIGO, International Federation of Gynecology and Obstetrics; ECOG, Eastern Cooperative Oncology Group; HR, Hazard ratio; CI, confidence intervals, ^indicates data is for both groups, * Used propensity score matching Continuous data as mean± SD or Median (interquartile range or range).

**Supplementary Table-II:** Adjusted factors and outcome data of included studies.

Study	Factors adjusted	HR (95% CI) for OS	HR (95% CI) for PFS
Abe 2010[36]	Surgical staging, histological cell type (serous and clear), residual tumor size, lymph node metastasis (pelvis and para-aortic), peritoneal cytology	NR	0.65 (0.23-1.61), 1.11 (0.77, 1.62)
Bao 2024[16]	FIGO pathological stage	0.969 (0.481-1.952)	1.110 (0.685-1.802)
Bizzari 2021[37]	Age > 60 years at diagnosis, serous histology, Grade 1, pathological stage I disease,	NR	0.52 (0.37-0.73)
Bizzarri 2023[14]	Age, lymphadenectomy	0.26 (0.08-0.80)	0.44 (0.19-1.02)
Chen 2021*[38]	Age, laterality, tumor size, race, histologic type, grade, apparent stage, number of dissected lymph nodes, chemotherapy	0.72 (0.40-1.30), 0.83 (0.48-1.43)	NR
Chen 2022[39]	Age, FIGO stage; debulking surgery, positive lymph nodes	5.90 (0.80, 43.59)	1.92 (0.38, 9.65)
Deng 2021[10]	Age, stage, histological grade, histological type, number of lymph nodes removed, region of lymphadenectomy, Lymph node metastasis	NR	0.01 (0.01 -1.12)
Ignatov 2022[40]	ECOG performance status, Age, adjuvant chemotherapy	1.15 (0.56-2.35)	0.87 (0.54-1.41)
Kim 2023[22]	Age, final stage, use of adjuvant chemotherapy	0.9 (0.3-2.8)	1.2 (0.5 to 3)
Liu 2024*[23]	FIGO stage, laterality, tumor size, CA-125 level, chemotherapy, and residual tumor	0.54 (0.17, 1.68)	0.46 (0.17, 1.25)
Maggioni 2006[13]	Treatment arm and grade	0.85 (0.49 - 1.47)	0.72 (0.46 - 1.21)
Matsuo 2018[15]	Age, race, tumor stage, histology types, tumor differentiation grade, tumor size	0.75 (0.68, 0.83)	NR
Michel 2023[24]	Age, Charlson score, histological type	0.59 (0.36-0.97)	NR
Miyamoto 2023*[25]	Age, CA-125, ascites>100ml, serous, chemotherapy	0.471 (0.094, 2.367)	0.684 (0.293, 1.595)
Nasioudis 2019[26]	Age, co-morbidities, insurance, disease sub-stage, tumor grade, tumor size	0.75 (0.62- 0.91)	NR
Oshita 2013[27]	Stage, histological type, adjuvant chemotherapy	0.71 (0.38, 1.13)	NR
Sun 2025[28]	Age, marital status, tumor size, grade, laterality, CA-125, stage, chemotherapy, surgery	0.697 (0.189, 2.57)	NR
Svolgaard 2014[29]	Comorbidity, cyst rupture, grade, peritoneal fluid cytology, performance score, final stage, histology	1.7 (0.9-3.0)	NR
Wang 2024[30]	Age, complication, FIGO stage, Pathological type, Chemotherapy, drop of CA125 value (≥0.5)	NR	1.687 (0.928-3.067)
Yamazaki 2018[31] R	Age, tumor size, node status, peritoneal cytology, capsule rupture, peritoneal involvement, combined chemotherapy	0.36 (0.17, 0.77)	0.41 (0.21-0.79)
Yang 2023[32]	Age, preoperative serum CA125 level, histologic grade, stage, fertility sparing surgery, chemotherapy	0.933 (0.234-3.714)	0.667 (0.326-1.367)
Yoshihara 2020*[33]	Age, body mass index, tumor size, CA-125 level at initial diagnosis, existence of preoperative capsule rupture and/or positive ascitic cytology, chemotherapy performance	0.960 (0.301, 3.062)	0.459 (0.083, 2.539)
Yoshihara 2021*[34]	Age, body mass index, presence of endometriosis, CA-125 level at the initial diagnosis, tumour size, uterine preservation, tumour stage, tumour grade, chemotherapy performance	0.895 (0.386-2.071)	NR
Zhao 2017[35]	Menopausal status, FIGO stage, histology grade, cytology of ascites, chemotherapy cycle	NR	0.18 (0.04-0.83)

One study^23^ revealed only PFS of mixed subtypes, whereas another^14^ reported both OS and PFS of endometrioid EOC. Precautions were taken to prevent data overlap within the same analysis. The majority of studies included a control group with “no lymphadenectomy.” Nonetheless, the control group in five of the investigations underwent partial staging or lymph node sampling. Many studies reported AC data, although the variation ranged from 19.4% to 100%. Sixteen studies provided data on lymph node metastases within the lymphadenectomy cohort, with rates varying from 1.8% to 22%. The factors adjusted by the studies when reporting outcomes and the median follow-up were inconsistent. Quality assessment carried out by two reviewers is shown in Supplementary Table-III. The majority of studies received a score of eight, while only three attained scores of six or seven. For the RCT, concerns were noted in the implementation and outcome assessment. Overall, quality was moderate.

**Supplementary Table-III:** Newcastle Ottawa scale score of studies.

Study	NOS breakdown S-selection C-comparability O-Outcome	Total
Abe 2010	S**** C** O**	8
Bao 2024	S**** C* O**	7
Bizzari 2021	S**** C** O**	8
Bizzarri 2023	S**** C- O**	6
Chen 2021*	S**** C** O**	8
Chen 2022	S**** C** O**	8
Deng 2021	S**** C** O**	8
Ignatov 2022	S**** C** O**	8
Kim 2023	S**** C** O**	8
Liu 2024*	S**** C** O**	8
Maggioni 2006	-	-
Matsuo 2018	S**** C** O**	8
Michel 2023	S**** C* O**	7
Miyamoto 2023*	S**** C** O**	8
Nasioudis 2019	S**** C** O**	8
Oshita 2013	S**** C** O**	8
Sun 2025	S**** C** O**	8
Svolgaard 2014	S**** C** O**	8
Wang 2024	S**** C** O**	8
Yamazaki 2018	S**** C** O**	8
Yang 2023	S**** C** O**	8
Yoshihara 2020*	S**** C** O**	8
Yoshihara 2021*	S**** C** O**	8
Zhao 2017	S**** C** O**	8

### Primary analysis:

**Fig.2:**
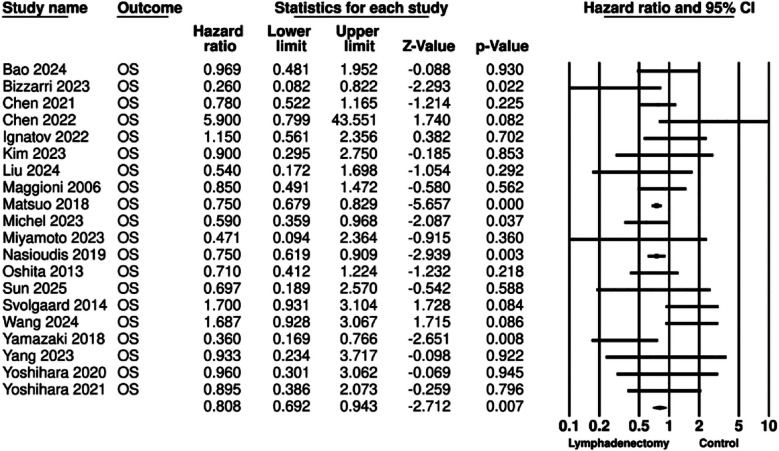
Meta-analysis of OS based on lymphadenectomy for early EOC.

**Supplementary Fig.1:**
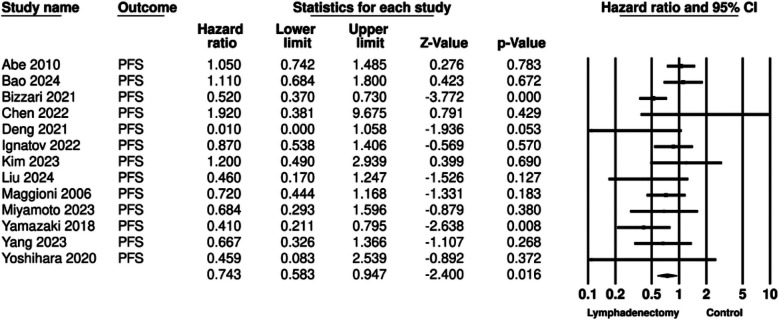
__________________________________.

**Supplementary Fig.2:**
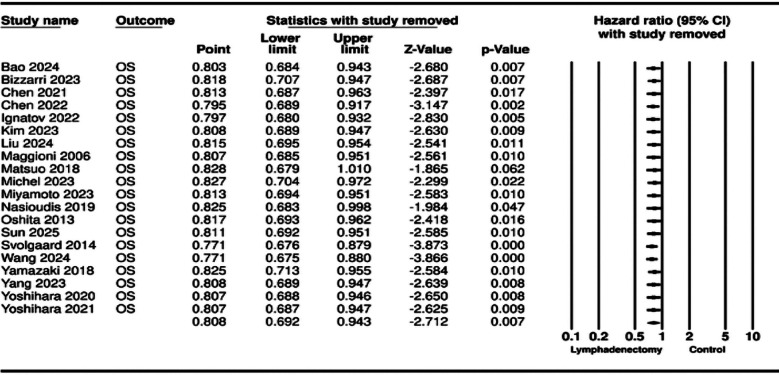
_____________________________.

The meta-analysis for OS included 20 studies. Aggregation of data revealed that patients undergoing lymphadenectomy had significantly better OS as compared to controls (HR: 0.808 95% CI: 0.692, 0.943 I^2^=34%) (Fig.2). Removing one study at a time in the sensitivity analysis failed to change the significance of the results for all except for one study of Matsuo et al^15^ (Supplementary Fig.1). No major asymmetry was noted on the funnel plot and Egger’s test showed no publication bias (p=0.42) (Supplementary Fig.2).

**Fig.3:**
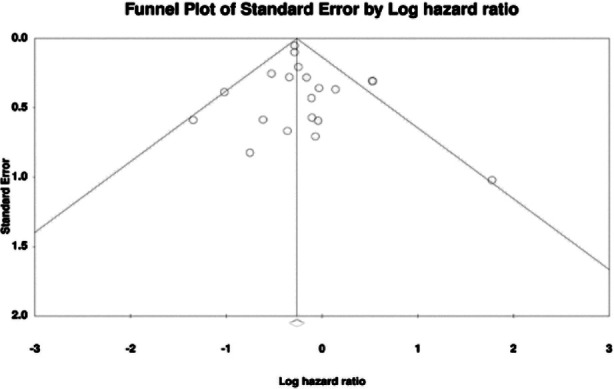
Meta-analysis of PFS based on lymphadenectomy for early EOC.

**Supplementary Fig.3:**
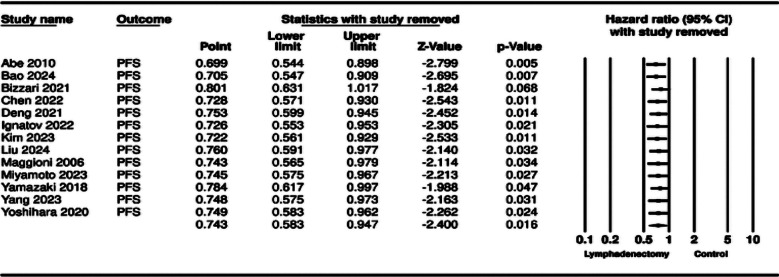
__________________________________.

**Supplementary Fig.4:**
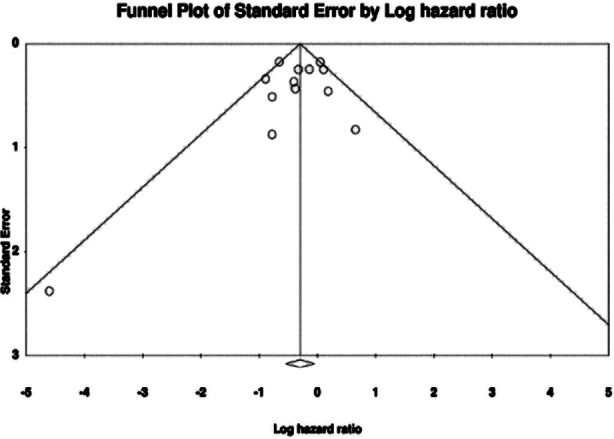
__________________________________.

The meta-analysis for PFS included 13 studies. Aggregation of data showed that that patients undergoing lymphadenectomy had significantly better PFS as compared to controls (HR: 0.743 95% CI: 0.583, 0.947 I²=44%) (Fig.3). Again, results were stable during sensitivity analysis for most studies except for Bizzari et al^37^ (Supplementary Fig.3). No major asymmetry was noted on the funnel plot and Egger’s test showed no publication bias (p=0.45) (Supplementary Fig.4).

### Subgroup and meta-regression analysis:

Outcomes of subgroup analysis can be found in Supplementary Table-IV. For OS, the results were non-significant in the prospective study and RCT, but significant in the retrospective data. Results were also rendered non-significant for studies conducted in Asia and Europe but not for North America. Histology-based sub-group analysis revealed significant results exclusively for clear cell EOC, with no notable findings in other subgroups. The results were significant solely for FIGO stages I-III, and not for studies that encompassed stage-I or stages I-II EOC. According to the type of lymphadenectomy, studies eliminating both or a single group of lymph nodes produced significant results; however, studies including both para-aortic and pelvic lymphadenectomy produced non-significant findings. Upon segregating studies according to control group protocol, results were not significant for those without lymphadenectomy, but were significant for those undertaking partial staging or lymph node sampling. Classifying studies according to the adjustment of AC and stage rendered the majority of results non-significant.

**Supplementary Table-IV:** Results of subgroup analysis.

Variable	Subgroups	Studies	Heterogeneity (I^2^)	HR (95% CI)
***Overall survival***				
Study type	Prospective	1	-	1.7 (0.89, 3.25)
	Retrospective	18	22	0.77 (0.67, 0.89)
	RCT	1	-	0.85 (0.49, 1.47)
Location	Asia	11	35	0.85 (0.63, 1.14)
	Europe	5	66	0.86 (0.62, 1.20)
	North America	4	0	0.76 (0.60, 0.97)
Histology	Mixed	11	40	0.87 (0.72, 1.04)
	Clear cell	2	0	0.41 (0.21, 0.80)
	Endometrioid	3	32	0.60 (0.32, 1.13)
	Mucinous	3	0	0.78 (0.55, 1.09)
	Serous	1	-	5.9 (0.78, 44.64)
Stage	I	8	2	0.86 (0.66, 1.13)
	I-II	11	44	0.81 (0.65, 1.00)
	I-III	1	0	0.26 (0.08, 0.87)
Lymphadenectomy	Both	7	32	0.75 (0.55, 1.02)
	Para-aortic and/or pelvic	11	0	0.78 (0.66, 0.93)
Control group	No lymphadenectomy	15	41	0.86 (0.69, 1.07)
	Lymph node sampling/partial staging	5	4	0.72 (0.53, 0.99)
Adjusted for chemotherapy	Yes	12	12	0.82 (0.64, 1.06)
	No	8	56	0.80 (0.65, 0.99)
Adjusted for stage	Yes	14	43	0.84 (0.70, 1.00)
	No	6	18	0.71 (0.51, 1.00)
***Progression-free survival***				
Study type	Retrospective	12	48	0.74 (0.57, 0.98)
	RCT	1	-	0.72 (0.33, 1.55)
Location	Asia	9	44	0.76 (0.55, 1.05)
	Europe	3	38	0.62 (0.45, 1.00)
	North America	1	-	1.2 (0.42, 3.42)
Histology	Mixed	8	51	0.78 (0.60, 1.01)
	Clear cell	2	0	0.43 (0.22, 0.82)
	Mucinous	2	0	0.95 (0.40, 2.28)
	Serous	1	-	1.92 (0.36, 10.33)
Stage	I	3	0	0.81 (0.40, 1.65)
	I-II	8	30	0.77 (0.50, 1.03)
	I-III	2	88	0.74 (0.44, 1.26)
Lymphadenectomy	Both	7	24	0.61 (0.48, 0.78)
	Para and/or pelvic	6	0	0.97 (0.74, 1.28)
Control group	No lymphadenectomy	10	48	0.80 (0.61, 1.06)
	Lymph node sampling/partial staging	3	0	0.56 (0.33, 0.94)
Adjusted for chemotherapy	Yes	7	0	0.67 (0.46, 0.97)
	No	6	67	0.81 (0.58, 1.15)
Adjusted for stage	Yes	9	61	0.74 (0.54, 1.01)
	No	4	0	0.74 (0.47, 1.18)

HR, hazard ratio; CI, confidence intervals.

Subgroup analysis of PFS also revealed similar results based on study type and histology with results significant only for retrospective studies and clear cell EOC. Segregation of studies based on location and stage led to non-significant results for all sub-groups. Lymphadenectomy showed significantly better PFS in studies wherein both group of lymph nodes were removed but not in studies removing both or one of the groups. Based on control group protocol, results were not significant for those without lymphadenectomy, but were significant for those undertaking partial staging or lymph node sampling. Most results were not significant on classifying studies based on adjustment of AC and stage.

Meta-regression analysis results are shown in Supplementary Table-V. It was seen that lymph node metastasis in the lymphadenectomy group and use of AC in the lymphadenectomy and control groups did not have a significant impact on the results.

**Supplementary Table-V:** Outcomes of meta-regression analysis.

Moderator	Beta co-efficient	95% Confidence Intervals	P-value
***Overall survival***			
Lymph node metastasis	0.0096	-0.0241 to 0.0433	0.58
AC in study group	0.0017	-0.0090 to 0.0124	0.75
AC in control group	0.0078	-0.0058 to 0.0215	0.26
***Progression free survival***			
Lymph node metastasis	-0.0205	-0.0581 to 0.0170	0.28
AC in study group	0.0001	-0.0160 to 0.0162	0.99
AC in control group	-0.0082	-0.0263 to 0.0098	0.37

## DISCUSSION

Compared to other gynecological malignancies, ovarian cancer frequently metastasizes to the pelvic and para-aortic lymph nodes, and sampling these nodes is crucial for disease staging.^8^-^10^ Lymphadenectomy is an intricate surgical technique associated with perioperative risks, including vascular and nerve damage, heightened blood loss, prolonged surgical duration, and a greater likelihood of lymphocele and lymphedema.^11^ Due to the limited incidence of lymph node invasion in eEOC, the significance of such an invasive surgery has been questioned, particularly regarding its impact on patient survival.^17^,^18^ Previously, Yao et al^17^ in a meta-analysis including four and three studies have shown that lymphadenectomy leads to improved OS (HR 0.78 95% CI 0.71, 0.86 I^2^=4%) and PFS (HR 0.62 95% CI 0.50, 0.78 I^2^=0%) in eEOC, respectively. Likewise, another meta-analysis by Yang et al^18^ showed similar results, wherein they noted significantly improved OS (HR: 0.72 95% CI: 0.61, 0.84 I^2^=0%) and PFS (HR: 0.74 95% CI: 0.67, 0.80 I^2^=38%) with lymphadenectomy in eEOC, but with only seven and eight studies respectively. Given the limited data available in both reviews, the conclusions are uncertain due to the small sample size (4-8 articles).

In the present study, we conducted an updated literature search and significantly increased the statistical power of the analysis to present the most comprehensive evidence on the impact of lymphadenectomy on survival after eEOC. Pooled analysis of data from 20 and 13 studies showed that lymphadenectomy was associated with a 20% and 26% improvement in OS and PFS, respectively. The results appeared robust in the leave-one-out analysis, exhibiting minimal variation in significance. Nevertheless, subgroup analyses based on several crucial variables revealed numerous non-significant outcomes, suggesting that various confounders continue to influence the association between lymphadenectomy and survival.

Of note, the results were significant only for retrospective studies and not for the prospective study^34^ or the RCT.^13^ The potential for selection bias in retrospective studies cannot be dismissed, and it is plausible that surgeon preference for performing a lymphadenectomy may have been affected by patient factors leading to improved survival. While the best quality evidence can be only provided by an RCT, the conduct of such a trial especially in cancer patients is difficult. The LION study^12^ was one such trial that reported no survival benefit of lymphadenectomy in patients with advanced EOC with macroscopically complete resection and clinically negative lymph nodes, dismissing the need for the procedure at least in advanced cases.

Nevertheless, due to a lack of high-quality evidence, the 2019 ESMO-ESGO consensus conference for ovarian cancer still considered lymphadenectomy as a standard surgical staging method for clinically eEOC but the level of evidence was IV, and 22.5% of the experts failed to reach a consensus.^41^ The sole RCT^13^ on eEOC investigating the effects of lymphadenectomy exhibited several limitations, including an imbalance in AC, as 90% lymph node-positive patients received AC vs 56% node-negative patients. Moreover, survival end-points were secondary outcomes in the trial and it was insufficiently powered to identify clinically significant differences. Nevertheless, a large RCT on eEOC is currently underway in South Korea and its findings may yield high-quality evidence on this vexing issue.^42^

The quality of lymphadenectomy differed among the studies, with some conducting both para-aortic and pelvic lymphadenectomy, while others performed only one of the two procedures. Subgroup analysis generated mixed results for these subgroups further complicating the results. The number of lymph nodes removed also varied across the studies, with several failing to report this data. Adequate dissection of at least 10 lymph nodes is the standard practice for staging eEOC.^43^ An adequate lymphadenectomy enhances the likelihood of diagnosing advanced-stage disease by increasing the number of nodes removed, thereby facilitating the identification of occult metastasis.^15^ Variation were also noted in the control group with five of the included studies conducting lymph node sampling. The study of Bizzari et al^37^ have compared lymph node sampling (removal of 1-19 nodes) and comprehensive staging (removal of >20 nodes) for eEOC only to find no significant difference in five years PFS (76.5% vs 79.7%) and OS (94.5% vs 92.3%) between the two modalities. Furthermore, both modalities were associated with superior survival outcomes as compared to no lymphadenectomy. Comprehensive retroperitoneal staging causes increased post-operative complications as compared to lymph node sampling alone and the latter may be preferred in clinical practice.^23^ Subgroup analysis showed that the HR were significant only for lymph node sampling studies but not for those without lymphadenectomy, perhaps due to the lower number of studies in the former. To provide a more accurate assessment of involved lymph nodes, use of sentinel lymph node biopsy has been suggested. It improves the identification of positive lymph nodes from a qualitative standpoint, rather than relying solely on the number of lymph nodes excised. Nonetheless, it remains in the trial phase for eEOC.^44^

Lymph node involvement in eEOC can vary with histologic subtype, reaching more than 10% in serous subtypes and generally remaining low for low-grade endometrioid or mucinous histology (<2%)^37,45^ For the clear cell subtype, the metastasis rate ranges from 0 to 11%.^28^ In our subgroup analysis, we were able to segregate data for some of these histological subtypes to note that lymphadenectomy offered a survival benefit only in clear cell eEOC, but not for endometrioid or mucinous histology. The results for serous eEOC were obtained only from a single study^25^ which included only low-grade tumors and hence must be interpreted with caution. Since most of the studies included mixed cases, the number of studies in the subgroups was very low. However, it can be suggested that lymphadenectomy should be employed as a diagnostic measure in patients at higher risk for lymph node metastases, but not in those with a very low incidence, as it has a limited role in assessing the need for AC in these instances.^46^

The role of AC in improving outcomes of eEOC cannot be underestimated. The EORTC-ACTION RCT has shown that use of AC was associated with superior recurrence-free survival in eEOC but the benefit was restricted to those without optimal staging.^47^ Thus, it may be considered that both comprehensive staging or AC can remove lymph node micro-metastasis in eEOC, and patients undergoing optimal lymphadenectomy can avoid AC.^32,48^ The use of AC varied in the included studies both in the study and control groups and subgroup analysis or meta-regression failed to demonstrate any clear conclusions. We believe the variations among studies reflects the current state of practice where choice of lymphadenectomy and AC is based on clinical factors like performance status, disease stage, histologic subtype, residual metastasis, etc. Lymphadenectomy may offers minimal diagnostic value in high-risk patients who are already planned for AC, irrespective of lymph node metastasis. However, lymphadenectomy can be considered for its diagnostic role in patients wherein AC is still not planned. Further studies segregating survival data post-lymphadenectomy based on receipt of AC are needed to provide more robust evidence.

### Limitations:

There are certain limitations to our review. Most studies were retrospective and likely influenced by selection bias. To address this issue, only adjusted outcomes were utilized for the analysis. Significant heterogeneity was observed in the studies regarding the adjusted covariates, and unaccounted confounders may have impacted the results. The inconsistency in cancer stage, histology, grade, therapeutic protocols—including surgical intervention and AC, and the definition of lymphadenectomy across the included studies may have contributed to fluctuations in the results. Lymphadenectomy-related complications could not be analyzed in our meta-analysis due to insufficient data. This variable significantly influences the clinical decision regarding the inclusion of complete staging in treatment plans. Future studies should report this data for a more thorough analysis. Finally, the definition of eEOC lacked rigorous standardization, and three articles included a limited number of stage III patients. More rigorous studies with large sample size, standardized treatment plans, and segregating data based on histology, stage, and receipt of AC are needed to verify the present results.

## CONCLUSIONS

The results of this updated meta-analysis indicate that lymphadenectomy is associated with improved OS/PFS in patients with eEOC, but the surgical risks and potential benefits need to be carefully weighed. Both OS and PFS seem to be improved with lymphadenectomy in eEOC. Nevertheless, the current evidence is based mostly on retrospective data with high degree of selection bias and heterogeneity and therefore must be interpreted with caution. According to ESMO-ESGO consensus guidelines and this review, we recommend that choice of lymphadenectomy should be based on individual patient’s age, risk of complications, and histological type, till high quality RCTs are published. A multicenter RCTs are needed in the future, especially focusing on the interaction between different histological subtypes and the scope of lymph node dissection.
